# For Perspiring Hands

**Published:** 1900-02-15

**Authors:** 


### FOR PERSPIRING HANDS.

Borax and salicylic acid, of each fifteen parts; boric acid, five parts; glycerin and proof spirit, of each sixty parts. Apply with rubbing, three times daily.—*Dental Brief.*

